# Fish peptone development using enzymatic hydrolysis of silver carp by-products as a nitrogen source in *Staphylococcus aureus* media

**DOI:** 10.1002/fsn3.198

**Published:** 2015-01-19

**Authors:** Meysam Fallah, Somayeh Bahram, Seyed Roholla Javadian

**Affiliations:** 1 Student of Fisheries, Qaemshahr Branch, Islamic Azad UniversityQaemshahr, Iran; 2 Department of Fisheries, Qaemshahr Branch, Islamic Azad UniversityQaemshahr, Iran

**Keywords:** Fish peptone, fish protein hydrolysate, silver carp by-products, *Staphylococcus aureus*

## Abstract

Fish peptone was produced using enzymatic hydrolysis of silver carp filleting by-products by alcalase and trypsin. Also, the efficiency of the hydrolysates as a nitrogen source in *Staphylococcus aureus* medium was compared with commercial TSB. The results indicated that the protein hydrolysate from alcalase and trypsin had high protein content (92.92%, 91.53 respectively), and degree of hydrolysis (4.94%, 4.6% respectively).The results showed that silver carp filleting waste can be an efficient source for fish peptone production as a nitrogen source for *S. aureus* medium. However, the type of the used proteolytic enzyme considerably affected the performance of the resulting peptone despite the same DH. Fish peptone produced by alcalese performed significantly (*P* < 0.05) better than commercial TSB as a media for the bacteria while the performance of the trypsin peptone was not as good as the commercial medium.

## Introduction

Seafood processing industry generates up to 60% by-products including head, skin, trimmings, fins, frames, viscera and roes, and only 40% fish products for human consumption (Dekkers et al. 2011). Fish filleting, salting, and smoking produce the major amount of solid by-products (50–75% of the fish) with a total of more than 3.17 million tons per year (Ferraro et al. 2010). Waste and by-products discarded by fisheries are currently increasing, driven by both a net increase in seafood consumption and the changing consumer trend toward ready-to-eat products. These large quantities of fish by-products require appropriate management, especially because they create serious pollution and disposal problems in both developed and developing countries. On other hand, these by-products are considered to be protein-rich material that is normally processed into low market-value products, such as animal feed, fish meal, and fertilizer (Hsu 2010). However, during the last decade, several biotechnological methods have been developed to recover these essential nutrients to prepare better use of this protein-rich resource and to solve the pollution and disposal problems as well. One of the efficient biotechniques that are currently employed is enzymatic hydrolysis of fish proteins that allows the production of a broad spectrum of food ingredients or industrial products for a wide range of applications, including fish peptones (Chalamaiah et al. 2012).

Peptones are a water-soluble mixture of polypeptides and amino acids which are widely used in many biological and biotechnological applications, such as microbial biomass. Peptones are primarily obtained from the products of bovine or porcine origin, such as meat, internal organs, gelatin, and milk, but also from plants and yeasts (Bridson and Brecker 1970). Because of recent outbreaks of bovine spongiform encephalopathy and a growing requirements for raw materials that are kosher approved and certified free of swine flu, peptones of a nonmeat origin are becoming increasingly important. Also, increasing demand for microbial growth media for the biotechnological fermentation industry has raised attention for new and inexpensive peptone sources because the nitrogen source made the most expensive part of microbial growth media (Aspmo et al. 2005a; Safari et al. 2012). Several investigation (Horn et al. 2000; Aspmo et al. 2005a,b; Gildberg et al. 2010; Safari et al. 2012) have shown that due to its favourable amino acid balance and high protein content, fish materials represent a potential source of industrial peptones. Nevertheless, no work has been reported on the use of fish peptone produced using enzymatic hydrolysis of silver carp filleting by-products (trimmings) using different enzymes as a nitrogen source in *Staphylococcus aureus* media.

The present work of the enzymatic hydrolysis of silver carp by-products is aimed primarily at the industrial application of the process. This poses constraints, particularly with respect to the overall cost efficiency of the scaled-up process. Low cost and simplicity in operation, by reducing the cost of material, energy consumption, important attributes that outline the direction of this work (Aristotelis et al. 2011).

Thus, the present work was aimed to develop fish peptone from silver carp filleting by-products using enzymatic hydrolysis with different enzymes and to evaluate their suitability as microbial growth media for *S. aureus*.

## Material and Methods

### Fish peptone preparation

Fresh silver carp were bought from a local aquaculture farm. In 1 h, they were transported to the laboratory in sealed foamed polystyrene boxes containing flaked ice. Then, the fish were gutted, skinned, filleted, and the by-products were gathered and washed (with tap water) by hand. Then, filleting by-products (trimmings and cut off) were minced twice at medium speed using an industrial mixer (5 mm blade size; Jaltajhiz, Tehran, Iran). The minced by-products were frozen at −20°C for further analysis. Proximate composition of the minced samples was determined within 2 days after freezing the mince by-products.

In order to hydrolyze the samples, the mixed by-products were thawed overnight at 10°C and mixed with distilled water (2:1w/v). Then, the mixture was heated at 85°C in a water bath (W614-B; Fater Rizpardaz, Tehran, Iran) for 20 min to inactivate the endogenous enzymes. Alcalase 2.4L and trypsin (Sigma Aldrich, Darmstadt, Germany) were added separately to the substrate at 1.5% (v/w) and 1.5% (w/w), respectively. Then, optimum conditions for hydrolysis were standardized for each enzyme which included pH (8.5 for alcalase and 7 for trypsin), temperature (55°C for alcalase and 37°C for trypsin). All reactions were conducted in triplicate in 250 mL glass Erlenmeyer flasks in a shaking incubator (GTSL20; Jaltajhiz) with constant agitation (at 150 rpm) for 2 and 4 h about alcalase and trypsin, respectively.

After passing the reaction time, the enzymes were thermally deactivated by heating at 95°C in a water bath for 20 min to terminate the reactions. Then, the hydrolyzed mixtures were centrifuged (6700*g*, 20 min) using 1.5-mL tubes at 10°C in a centrifuge (D-7200; Hettich, Tuttlingen, Germany). Finally, the supernatants were collected to obtain the soluble peptones for further experiments (Safari et al. 2011), then stored at −20°C, until they were freeze dried.

### Bacterial strain and maintenance

*S. aureus* was used in the present study. The bacterium was purchased from the Iranian Research Organization for Science and Technology (IROST), Iran. The strain PTTC was transferred to tryptic soy broth (Merck, Darmstadt, Germany) and it was incubated at 30°C for 12–18 h and then these appropriately prepared cultures were used for further experiments (Vazquez et al. 2004).

### Bacterial growth media and culture

Bacterial media compositions were prepared according to Safari et al. (2012) and they are shown in Table1. The TSB medium was used as control (commercial medium) and the proteinacous compounds in the TSB medium were replaced by the peptones from the hydrolyzed trimming by-products for other treatments. Then, the initial pH of the obtained medium was adjusted to 7.3 using 0.2N NaOH, and the solutions were sterilized at 121°C for 15 min at a pressure of 1.1 atm. The bacteria was cultured in 250-mL Erlenmeyer flasks containing 150 mL of different mediums in triplicate, at 30°C with a 150 rpm shaking speed in an incubator. The medium had been inoculated with *S. aureus* at 3% (v/v) from 18 h aged cultures on TSB medium, adjusted to an OD (*λ* = 600) of 0.50 using a UV-visible spectrophotometer (Eppendorf, Hamburg, Germany).

**Table 1:** Composition of culture media used in the microbiological tests (G/L)

Ingredient	Fish peptone media	TSB media
Sodium chloride	5.00	5.00
Peptone from casein	–	15.00
Peptone from soymeal	–	5.00
Silver carp peptone (Biuret)	20.00	–

Bacterial density in different media was determined by measuring turbidity at 3-h intervals at *λ *= 600 nm using the spectrophotometer. All culture media were lightly shaken for 5 sec before sampling to determine the OD at 600 nm.

### Chemical analysis

The degree of hydrolyzed (DH) protein was determined according to Hoyle and Merritt (1994) as described by Safari et al. (2012). After the specified hydrolysis, 20% trichloroacetic acid (TCA) was added to each treatment to terminate the reaction. Then, the solutions were centrifuged to collect the 20% TCA soluble material as the supernatant. After that, DH was calculated as follows:

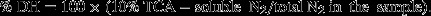
The Biuret method (Layne 1957) was used to determine protein content in the by-product hydrolysates in the supernatant following centrifugation. Bovine serum albumin was used as the standard protein (Zistchimi, Tehran, Iran).

### Statistical analysis

The differences among all measurements were evaluated by one-way analysis of variance (ANOVA). Duncan's multiple range tests were used to compare the means to identify which groups were significantly different from other groups. Significance was defined at *P *<* *0.05. All data are presented as mean ± SD.

## Results and Discussion

### Chemical composition

Total protein and fat content of silver carp filleting by-products were 17.89 ± 0.20 and 2.53 ± 0.7, respectively. Although the composition of fish products and by-products depends on nutrition, fish size, sex, age, environment, and season, the obtained results coincide with others (Abdollahi et al. 2014). However, body composition changes greatly from one species to another and one individual to another. Thus, notable variations may be observed in the components of fish muscle (Pacheco-Aguilar et al. 2000).

Chemical characteristics of peptones obtained by hydrolysis of silver carp by-products are shown in Table2. As can be seen, the DH of the peptones produced by alcalase and trypsin were 4.94% and 4.60%, respectively. Although the DH of the peptones produced by alcalase was higher than the other one, they were not significantly different (*P* > 0.05). The hydrolysis of by-product protein with both enzymes proceeded at a high rate during the initial 20 min and slowed down thereafter. It means that the maximum hydrolysis of peptide bonds has been done within the initial 20 min of hydrolysis. Thus, the hydrolysis was continued for a maximum time of 2 and 4 h in the present study. Previous studies have also reported the same trend for enzymatic hydrolysis of fish proteins (Batista et al. 2009). Higher DH observed for hydrolysates produced by alcalase compared to trypsin was also reported by Ovissipour et al. (2009) who compared the effect of four different enzymes (alcalase, trypsin, protamax, and neutrase) in the hydrolysis of Persian sturgeon viscera. The same results were also reported about fish peptone produced from tuna head (Safari et al. 2012). However, the application of different hydrolysis time caused the production of protein hydrolysates with approximately equal DH for both enzymes.

**Table 2:** Chemical characteristics of silver carp by-product peptones

Name	DH%	Ash%	Soluble protein%	Protein (dry matter)%
Alcalase	4.94 ± 0.15a	3.50 ± 0.10a	37.01 ± 0.20a	92.92 ± 0.18a
Trypsin	4.60 ± 0.38a	3.7 ± 0.21a	26.49 ± 0.40b	91.53 ± 0.19a

Values in column with different letter are significantly different at α= 0.05

The soluble protein of the peptone obtained by alcalase was higher than the peptone produced by trypsin. The results were in agreement with results reported by others about alcalase efficiency in fish by-product hydrolysis (Ovissipour et al. 2009). However, Reissbrodt et al. (1995) mentioned that to evaluate peptone efficiency in growth media, physical and chemical data alone are not sufficient to understand the overall effect on microbial growth. Regarding the fact that peptones in microbial media provides the main sources of nitrogen and carbon, a successful peptone source is better evaluated by the growth of the organism of interest (Vieira et al. 2005).

### Microbial growth curve

**Figure 1:**
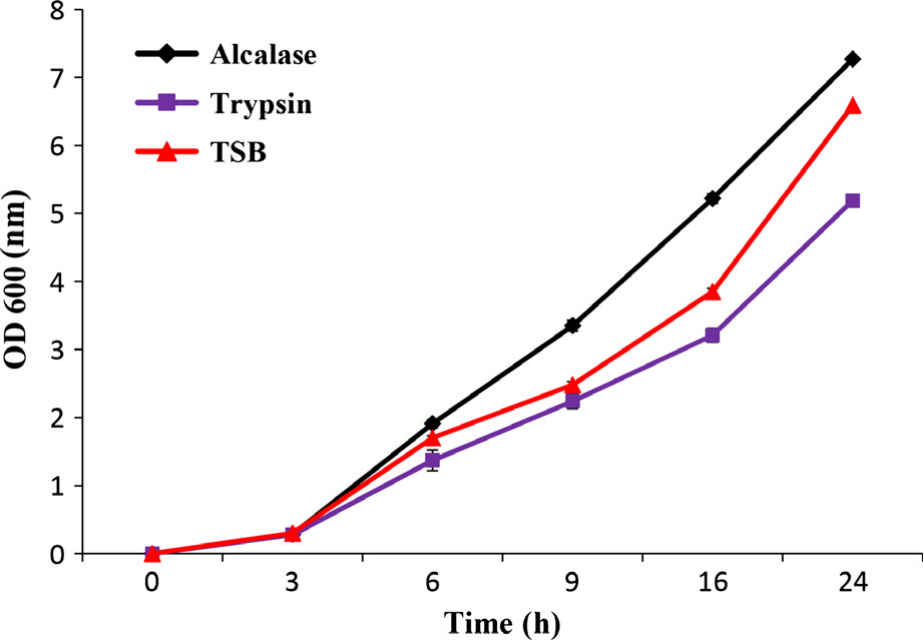
Bacterial growth curves monitored as optical density.

The growth curves of *S. aureus* on three different media are shown in Figure1. As can be seen, the bacteria grew properly in all three media. However, the best growth rate was observed for the bacteria in the medium containing alcalase peptone compared to TSB and trypsine medium which showed significantly higher OD during the study period (*P* < 0.05). The better results obtained for alclase peptone may be related to the fact that alcalase leads to optimal uptake of the available amino acid resources because this endoprotease's activity is most complementary to the bacteria's proteolytic and peptide-uptake systems (Safari et al. 2012). Also, Aspmo et al. (2005b) showed that alcalase produced peptides having a higher content of short peptides. Another possible explanation may be that the hydrolysates made with alcalase have shown a decrease in high-molecular weight fractions and increased solubility (Kristinsson and Rasco 2000). Peptones produced from different fish sources (tuna, cod, salmon, and unspecified fish) were compared with a casein peptone and results for six species of bacteria, yeasts, and fungi demonstrated in most cases the efficiency fish peptones (Dufosse et al. 2001). Vieira et al. (2005) also reported superior performance of peptones produced from different fisheries by-product for *Escherichia coli* compared to commercial peptone. The better performance of fish peptone in comparison with commercial media was also reported in other researches (Vieira et al. 2005; Safari et al. 2011, 2012). Moreover, other studies introduced alcalase as a better enzyme for fish peptone production from cod viscera for *E. coli* (Aspmo et al. 2005b) and *Lactobacillus plantarum* (Horn et al. 2000) and yellow fin tuna head for seven LAB strains (Safari et al. 2012) compared to other enzymes. They related their results to a higher degree of hydrolysis in peptones from alcalase which would accelerate the absorption of peptones from the medium (Safari et al. 2012) while in this study both peptones had similar DH.

On the other hand, in the present study, *S. aureus* showed lower growth rate with fish peptone medium produced by trypsin in comparison with TSB. It may be related to the differences between peptide chain lengths in peptones produced by different enzymes.

## Conclusions

The present study reveals that silver carp filleting by-product can be an efficient source for fish peptone production as a nitrogen source for *S. aureus* medium. However, the efficiency of the peptones as microbial growth depends on the enzyme type used for hydrolysis. Fish peptone produced by alcalese performed better than commercial TSB as a medium for the bacteria while the performance of the trypsin peptone was not as good as the commercial medium.

## Conflict of Interest

None declared.
